# Supplementary material for: Changes in elbow flexion EMG morphology during adjustment of deep brain stimulator in advanced Parkinson’s disease
Source: PLoS One. 2022 Apr 14;17(4):e0266936. doi: 10.1371/journal.pone.0266936 (PMC9009623; doi:10.1371/journal.pone.0266936)
Supplement: Attachment — Submitted filename: Elbow flexion_180621.docx [file pone.0266936.s002.docx]

**General comments**

This manuscript investigates the effect of modifying DBS settings on EMG of the biceps brachii muscle bilaterally and the UPDRS III clinical sub-scores for tremor and rigidity. Results indicate that whilst clinical sub-scores did not change between settings, there were significant differences in EMG metrics. This is an interesting and relevant study to better understand if and how motor changes relate to modifying DBS frequency, width, and amplitude settings. There are however several general areas that need to be addressed:

- There are numerous grammatical errors throughout the manuscript usually with a word missing, incorrectly used word and several typos. Please proofread the manuscript and correct.
- No discussion of how altered ‘EMG signal morphology’ relates to clinical changes has been made. Is there any evidence that for example increased recurrence rate (greater repetition) relates to improved clinical function?
- Complexity has a definite mathematical definition and indicates greater interactions (Didier Delignieres & Vivien Marmelat, Fractal Fluctuations and Complexity: Current Debates and Future Challenges, December 2012, Critical Reviews in Biomedical Engineering 40(6):485-500). Decreased regularity does not mean increased complexity. Please check throughout the manuscript that the use of the word ‘complexity’ is appropriate.
- Tremor and rigidity were assessed with sub-scores of UPDRS III which is a very blunt tool. Tremor can be measured quantitatively with an accelerometer and an indication of rigidity by measuring co-activation of agonist and antagonist. Why were quantitative measures of recording tremor and rigidity not undertaken?
- Statistics is only very briefly mentioned. Only a Wilcoxon signed rank test was performed. Were the data nonparametric? Could Friedman/ Kruskal Wallis be used? Please expand more on the statistics.

**Methods**

- In Methods, how was the location of placement of the electrodes determined? Were SENIAM guidelines followed?
- At what angle was the elbow joint at the beginning of the recording session and what was the angular range of movement and velocity? Length of fascicle, angular velocity and eccentric/ concentric contractions will all modify EMG signals.
- Please include the safety levels for the DBS parameters.
- Please provide a reference on pg4, line 124 for why 5 minutes was selected between setings.

**Analysis**

- How were the flexion phases of the EMG signals selected?
- Please explain why kurtosis, recurrence rate and correlation dimension were analysed and not other metrics. What algorithms were used and what software?
- **Results**
- A demographics’ table with individual’s demographics and clinical details should be included

**Discussion**

- Line 217 – Is it possible for anything to change ‘instantly’? Please revise.
- ‘Miniscule’ is a vague term and preferable to be replaced with ‘nonsignificant changes’.
- Lines 238-240. EMG records muscle activity therefore by definition it will have greater sensitivity to muscle activity changes as this is what it is measuring! Please revise. EMG is quantitative and UPDRS III is a subjective qualitative clinical measure.
- Line 240 – How is ‘optimally tuned’ DBS defined?

**Conclusion**

- The conclusion is weak as there is no strong message other than changes occur. It is recommended to introduce some clinical relevance.

**Figures**

Figure 1- Please either define the acronyms of the 7 settings in the figure legend or ideally, add as subheadings.

Figure 2 – See above.
